# Effects of virtual reality-based cognitive training on cognitive function, instrumental activities of daily living, and depressive symptoms in older people with mild cognitive impairment: a systematic review with meta-analysis of randomized controlled trials

**DOI:** 10.3389/fneur.2026.1728495

**Published:** 2026-04-13

**Authors:** Edgar Vásquez-Carrasco, María Sepúlveda-Ramirez, Yuslavia Alegria, Macarena Benavides, Jordan Hernandez-Martinez, Eduardo Fernández-Rodríguez, Eduardo Carmine-Peña, Constanza Lorca, Cristian Sandoval-Vásquez, Pablo Valdés-Badilla

**Affiliations:** 1 School of Occupational Therapy, Faculty of Psychology, Universidad de Talca, Talca, Chile; 2 Centro de Investigación en Ciencias Cognitivas, Faculty of Psychology, Universidad de Talca, Talca, Chile; 3 VITALIS Longevity Center, Universidad de Talca, Talca, Chile; 4 Programa de Doctorado en Psicología, Universidad Católica del Maule, Talca, Chile; 5 Department of Physical Activity Sciences, Universidad de Los Lagos, Osorno, Chile; 6 Department of Education, Faculty of Humanities, Universidad de La Serena, La Serena, Chile; 7 Department of Developmental and Educational Psychology, University of Salamanca, Salamanca, Spain; 8 Carrera de Medicina, Facultad de Medicina, Universidad de La Frontera, Temuco, Chile; 9 Departamento de Ciencias Básicas, Facultad de Medicina, Universidad de La Frontera, Temuco, Chile; 10 Carrera de Terapia Ocupacional, Facultad de Ciencias de la Salud, Universidad Autónoma de Chile, Temuco, Chile; 11 Departamento de Medicina Interna, Facultad de Medicina, Universidad de La Frontera, Temuco, Chile; 12 Department of Physical Activity Sciences, Faculty of Education Sciences, Universidad Católica del Maule, Talca, Chile; 13 Sports Coach Career, Faculty of Life Sciences, Universidad Viña del Mar, Viña del Mar, Chile

**Keywords:** aged, cognition, older adults, rehabilitation, technology

## Abstract

**Background:**

This systematic review and meta-analysis aimed to synthesize and critically evaluate the available scientific evidence on the effects of virtual reality based cognitive training on cognitive function, instrumental activities of daily living (IADL) performance, and depressive symptoms in older peoples with mild cognitive impairment (MCI).

**Methods:**

A systematic review and meta-analysis of RCTs was conducted following PRISMA guidelines. Seven electronic databases (PubMed, EBSCOhost, CINAHL Complete, Cochrane, ProQuest, Scopus, and Web of Science) were searched through March 2026. Additional sources included the reference lists of relevant studies. Study quality was assessed using the Oxford Centre for Evidence-Based Medicine scale, RoB 2, and GRADEpro tools. Eligible studies included RCTs that evaluated immersive and non-immersive VR-based cognitive training interventions in older peoples with MCI, reporting outcomes on cognitive function, IADLs, or depressive symptoms. The review was registered in PROSPERO (CRD42024558108).

**Results:**

Of 2,433 screened records, 19 studies met the inclusion criteria. Pooled analyses revealed significant effects of VR-based cognitive training on cognitive function (Montreal Cognitive Assessment: *p* < 0.003; Trail Making Test–B: *p* < 0.000), IADLs (*p* < 0.000), and depressive symptoms (Geriatric Depression Scale: *p* = 0.002).

**Conclusion:**

VR-based cognitive training was associated with improvements in cognitive performance, instrumental activities of daily living, and depressive symptoms in older people with mild cognitive impairment. However, these findings should be interpreted with caution due to heterogeneity across studies, mixed risk of bias, and the moderate certainty of the evidence.

**Systematic review registration:**

https://www.crd.york.ac.uk/PROSPERO/view/CRD42024558108, identifier (CRD42024558108).

## 1 Introduction

Population aging has led to an increase in life expectancy and the proportion of older people, with projections estimating that by 2050, they will comprise 22% of the total population (1). Consequently, the prevalence of chronic diseases and conditions affecting quality of life is also rising (2), including mental health disorders, which affect 14% of older people and account for 10.6% of years lived with disability (3). In this context, mild cognitive impairment (MCI) is considered a transitional stage between normal aging and dementia (4), characterized by deficits in attention, executive functions, memory, language, and perceptual abilities, without an identifiable cause (5).

Interventions that combine physical activity, cognitive stimulation, and nutritional strategies have been shown to enhance neuroplasticity by promoting synergistic effects on brain structure and function (6). These approaches support cognitive resilience through mechanisms such as increased synaptic efficiency, improved cerebral perfusion, and modulation of neurotrophic factors, thereby contributing to the maintenance of cognitive function and functional independence in aging populations (7, 8). Cognitive stimulation has demonstrated improvements in various cognitive functions and a reduced risk of MCI (9, 10). Previous cognitive training modalities often lack and real-world applicability, whereas VR interventions simulate functional tasks more closely resembling daily life and have demonstrated significant improvements in global cognition compared with control conditions in older peoples with MCI (11). Additionally, when applied in group settings, it promotes psychosocial well-being and lowers the risk of depression and anxiety (12). Although instrumental activities of daily living (IADL) are globally preserved, subtle deficits emerge in high-demand tasks that may predict later decline, therefore, IADL are sensitive to changes in cognitive function (13). Depressive symptoms, common in MCI, are associated with worse cognitive performance and a higher risk of progression; their measurement captures the affective dimension that influences autonomy and prognosis (14). Jointly assessing cognitive function, IADL, and depression offers a comprehensive clinical view to evaluate the effectiveness of interventions (13, 14). Various meta-analyses report that virtual reality (VR) interventions significantly improve the reduction of depressive symptoms in older people with and without MCI (15, 16). Despite promising cognitive and functional benefits, VR interventions for older adults are limited by technological barriers, including usability challenges, variable immersion systems, and the need for standardized, accessible software to enable broader adoption and effective implementation (17). Furthermore, longer intervention durations were significantly associated with greater reductions in depression (18). One of the most novel therapeutic strategies in cognitive training is VR, which has been developed to mitigate the cognitive and functional impairments associated with neurocognitive disorders such as Parkinson’s disease, stroke, Alzheimer’s disease, and traumatic brain injury (19, 20). VR operates by recreating realistic environments, including social or domestic experiences, with varying levels of immersion through full, partial, or minimal interaction (21). Immersive VR uses head-mounted displays and motion tracking to create a strong sense of presence, while non-immersive VR delivers virtual environments through standard screens with lower immersion (22). Using devices that provide multisensory feedback, VR allows users to engage with simulated scenarios that closely resemble real-world experiences (23, 24).

A systematic evaluation by Zhong et al. (25) demonstrated that VR-based cognitive training significantly improves overall cognitive performance in older people with MCI. The results indicate that VR enhances skills including planning, organizing, and problem-solving in relation to IADL, hence improving participants’ functional capabilities. Yan et al. (26) assert that immersive experiences may mitigate cognitive decline, as VR training replicates real-life situations in a regulated setting, thereby improving skill transfer. Identifying aspects such as intervention intensity, ideal timing, and the need for standardized methods and systems is crucial (11). Various meta-analysis report that VR interventions significantly improve the reduction of depressive symptoms in older people with and without MCI (15, 16). Furthermore, longer intervention durations were significantly associated with greater reductions in depression (18). VR functions as an innovative tool with several uses in intervention, leisure, and recreation, offering new opportunities to improve independence in activities of daily living for older people with MCI (27). Given the current findings, as well as the heterogeneity in interventions, sample sizes, and dosages, this systematic review and meta-analysis aimed to compile and analyze the available scientific evidence regarding the impact of VR-based cognitive training on cognitive function, IADL performance, and symptoms of depression in older people with MCI.

## 2 Methods

### 2.1 Protocol and registration

This systematic review with meta-analysis followed the methodologies set forth by the Cochrane Collaboration (28) and conformed to the PRISMA checklist and flowchart stipulations for reporting (29). The review procedure has been documented in the PROSPERO database under the identification code CRD42024558108.

### 2.2 Eligibility criteria

This systematic review with meta-analysis included peer-reviewed original articles, specifically randomized controlled trials (RCTs), without restrictions on language or publication date, up to March 16, 2026. Exclusions included conference abstracts, books, book chapters, editorials, letters to the editor, protocol records, reviews, case studies, and non-randomized trials. The study selection was guided by the PICOS (Population, Intervention, Comparator, Outcome, Study Design) paradigm, as outlined in Table 1.

**Table 1:** Selection criteria used in the systematic review.

Category	Inclusion	Exclusion
Population	Studies were included if they involved people mean aged 60 years or older, with a diagnosis of MCI.	Studies with populations whose main pathology is other than MCI (chronic diseases, physical deterioration or social problems).
Intervention	Studies involving immersive or non-immersive virtual reality–based cognitive training interventions in older people with MCI, lasting 4 weeks or more, were considered eligible. Interventions were included when the virtual or digital interface was central to the delivery of cognitive training, even if the format varied in terms of immersion level or technological platform.	Studies that include other types of complementary interventions, not related to virtual reality.
Comparison	Interventions with active or inactive control groups.	Absence of control group.
Outcomes	At least one assessment of cognitive function, IADL and Depressive Symptoms.	Lack of baseline data and/or follow-ups.
Study design	Randomized controlled trials, with pre- and post-assessment.	Controlled, retrospective, prospective and cross-sectional, non-randomized studies.
Level of evidence	1a	1b, 2a, 2b, 3a, 3b, 4 and 5.

IADL, Instrumental Activities of Daily Living; MCI, Mild Cognitive Impairment.

### 2.3 Information and database search process

Seven databases were used: Medline/PubMed, Scopus, Cochrane, Web of Science (Core Collection), EBSCOhost, CINAHL, and ProQuest. Medical Subject Headings (MeSH) from the US National Library of Medicine and free-text phrases related to VR Cognitive Training, Cognitive Function, IADL, Depressive Symptoms, older people, and MCI were used. The following search string was applied (e.g., in PubMed): (“Cognitive Dysfunction” OR “Neurocognitive Disorders” OR “Cognitive Impairment Syndrome” OR “Early Cognitive Decline” OR “Mild Cognitive Changes” OR “Minor Cognitive Impairment”) AND (“Cognitive Training” OR “Psychomotor Performance” OR “Neuropsychological Tests” OR “Cognition” OR “Executive Function” OR “Brain Function” OR “Cognitive Process” OR “Cognitive Processes” OR “Cognitive Processing” OR “Cognitive Performance” OR “Cognitive Function” OR “Cognitive Functions”) AND (“Virtual Reality” OR “Virtual Reality Exposure Therapy” OR “Exergaming” OR “User-Computer Interface” OR “Augmented Reality” OR “Virtual Reality Immersion Therapy” OR “Virtual Reality Therapy” OR “Virtual Systems” OR “Immersive Virtual Reality” OR “Non-Immersive Virtual Reality” OR “Social Virtual Reality”) AND (“Independent Living” OR “Activities of Daily Living” OR “Oral Hygiene” OR “Hand Hygiene” OR “Eating” OR “Sexual Behavior” OR “Daily Living Activities” OR “ADL” OR “Daily Living Activity”) AND (“Aged” OR “Older Adults” OR “Older People” OR “Older Subject” OR “Aging” OR “Ageing” OR “Aged”). The included articles and the inclusion/exclusion criteria were reviewed by two independent experts with the following qualifications: (i) a Ph. D. in health-related sciences and (ii) peer-reviewed publications in journals with an impact factor (Journal Citation Reports®). The experts were not provided with the search strategy to minimize bias. A final database search on December 30, 2025, aimed to identify relevant errata or retractions related to the included studies.

### 2.4 Study selection and data collection process

The studies were exported to Mendeley Reference Manager (version 2.116.2), and the study selection process is illustrated in the PRISMA flowchart. Three authors (M. S.-R., Y. A. and M. B.) independently conducted the searches, systematically reviewing titles, abstracts, and full texts, while duplicates were removed. During the title/abstract and full-text screening phases, discrepancies between reviewers were identified. These discrepancies were resolved through discussion and consensus. Inter-rater agreement was assessed using Cohen’s kappa statistic, demonstrating a high level of agreement between reviewers. In cases where consensus could not be initially achieved, studies were re-evaluated jointly until agreement was reached.

### 2.5 Methodological quality assessing

Study design and level of evidence were classified according to the Oxford Centre for Evidence-Based Medicine framework (30). In addition, study-level methodological limitations were independently assessed using the RoB 2 tool, thereby providing a more rigorous evaluation of internal validity beyond design-based evidence classification alone (28).

### 2.6 Data collection process

Data from included studies were extracted into a standardized form using Microsoft Excel® (version 16.81), following Cochrane guidelines (31). Three researchers (M. S.-R., Y. A. and M. B.) performed extractions independently, comparing their results to ensure accuracy. Oversight was provided by a fourth author (E. V.-C.). Variables extracted included authors, country, study design, sample size, groups (*n*), mean age (years), type of intervention and control group, training volume (frequency, duration, intensity), cognitive function (assessments), IADL (assessments), depressive symptoms (assessments) and main outcomes.

### 2.7 Risk of bias

The risk of bias in the RCTs was assessed using the RoB 2 tool (28). Three reviewers (M. S.-R., Y. A. and M. B.) conducted the initial analysis, which was reviewed by two additional authors (E. V.-C. and J. H.-M.). Discrepancies were resolved through examination and consensus.

### 2.8 Meta-analysis measures

The study used a meta-analytic approach, with detailed methodology registered in PROSPERO (CRD42024558108). To analyze the data, pooled effect sizes were calculated as standardized mean differences (SMDs) using Review Manager software (RevMan, version 5.4). A *p*-value of less than 0.05 was considered statistically significant (32). Meta-analyses were conducted to compare post-intervention values between experimental and control groups for outcomes related to cognitive function, instrumental activities of daily living (IADL), and depressive symptoms. Random-effects models, using the DerSimonian–Laird method, were applied when substantial clinical or methodological heterogeneity was expected or observed (33). This approach assumes that true intervention effects may vary across studies due to differences in factors such as intervention type, duration, or participant characteristics. Results were pooled when at least three studies reported sufficiently comparable post-intervention outcomes (34). Statistical heterogeneity was assessed using Cochran’s Q test (35) and the I^2^ statistic, with I^2^ values of <25%, 25–50, and >50% indicating low, moderate, and high heterogeneity, respectively (33). Egger’s regression analysis was also performed to explore small-study effects and potential publication bias (36). Means, standard deviations (SDs), and sample sizes (n) were extracted for both experimental and control groups at the post-intervention assessment, allowing the calculation of pooled standardized effect sizes for the meta-analysis. Only outcomes reported in at least three independent studies were included in the meta-analysis. This criterion was used to ensure sufficient overlap and comparability across trials. When studies reported multiple measures within the same clinical domain, only outcomes meeting this threshold were eligible for quantitative synthesis, whereas additional measures reported in fewer studies were summarized narratively. This approach also helped avoid double counting of participants within pooled analyses. Sensitivity analyses were performed to examine the stability of the pooled estimates and to explore whether specific studies disproportionately contributed to the observed heterogeneity.

### 2.9 Certainty of evidence

The certainty of evidence from the included studies was evaluated using the Grading of Recommendations, Assessment, Development, and Evaluation (GRADE) framework (28, 37). Evidence was categorized as high, moderate, low, or very low. All analyses initially started with a high certainty, given the inclusion of RCTs, but were downgraded if concerns arose regarding risk of bias, consistency, accuracy, precision, transparency of results, or publication bias. Three reviewers (M. S., Y. A. and M. B.) conducted independent assessments, with disagreements resolved by consensus involving a fourth author (E. V.-C.).

## 3 Results

### 3.1 Study selection

**Figure 1:**
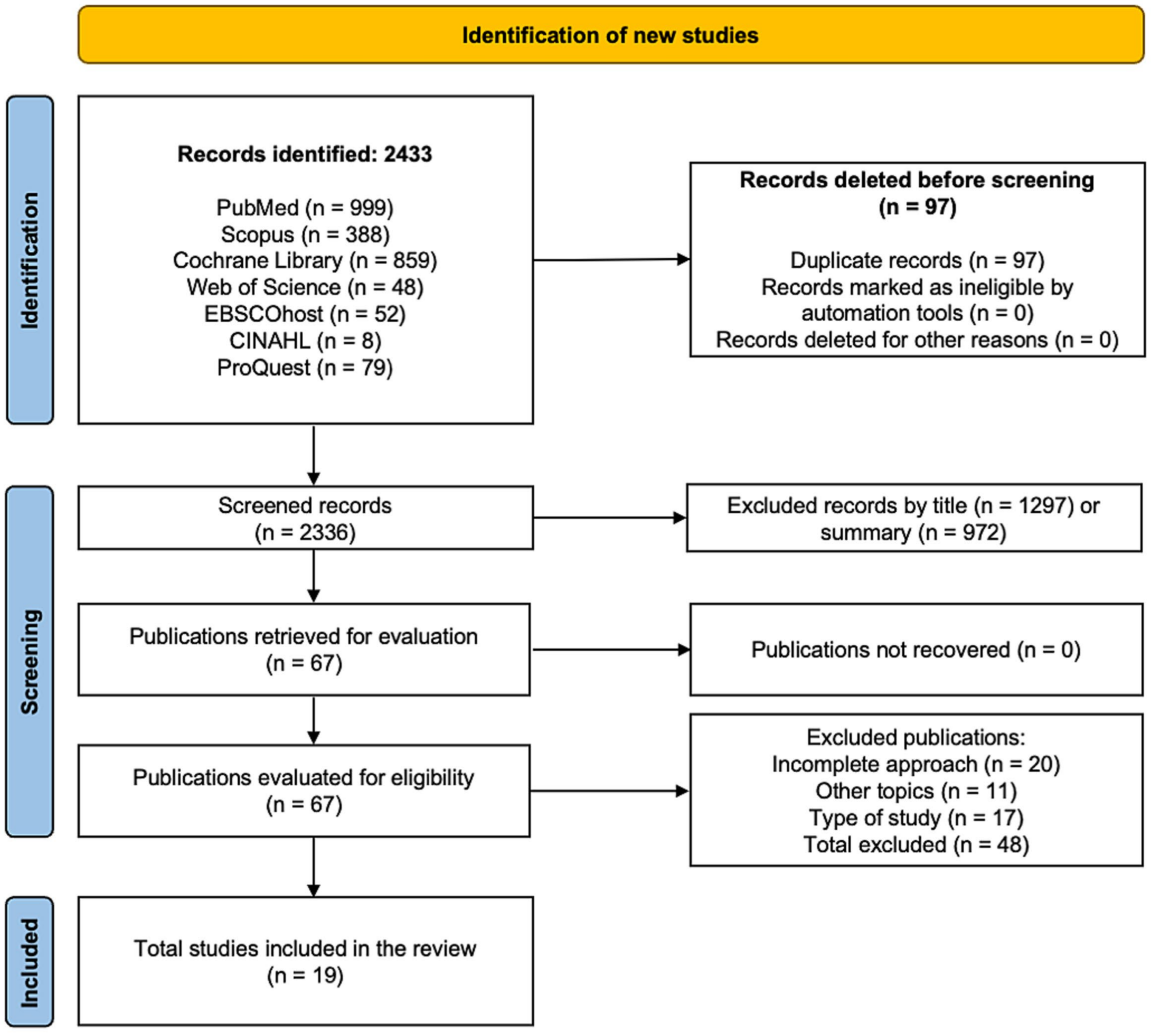
Flowchart of the systematic review.

A total of 2,433 records were identified through the database search, of which 97 were removed as duplicates. Of the remaining 2,336 records, 2,269 were excluded after title and abstract screening (1,297 based on title and 972 based on abstract). The full texts of 67 articles were then assessed for eligibility, and 48 were excluded for not meeting the predefined inclusion criteria: 20 did not evaluate a virtual reality–based cognitive training intervention with a clearly identifiable cognitive component, 11 involved populations, conditions, or research questions outside the scope of the review, and 17 did not meet the required study design because they were not randomized controlled trials. Finally, 19 studies were included in the systematic review and meta-analysis (38–56). The search results are presented in a flowchart according to PRISMA guidelines (57), as shown in Figure 1.

### 3.2 Methodological quality

All included studies were randomized controlled trials, corresponding to level 1b evidence according to the Oxford Centre for Evidence-Based Medicine; however, study design alone does not necessarily imply high methodological quality.

### 3.3 Risk of bias

**Figure 2:**
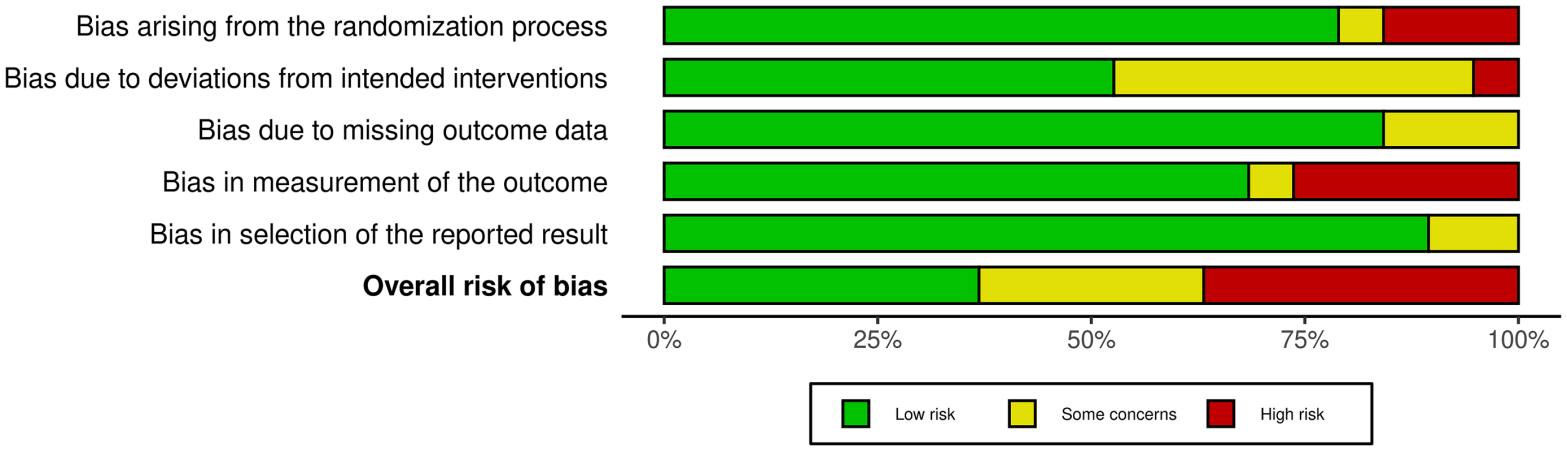
Summary of the risk of bias assessment.

**Figure 3:**
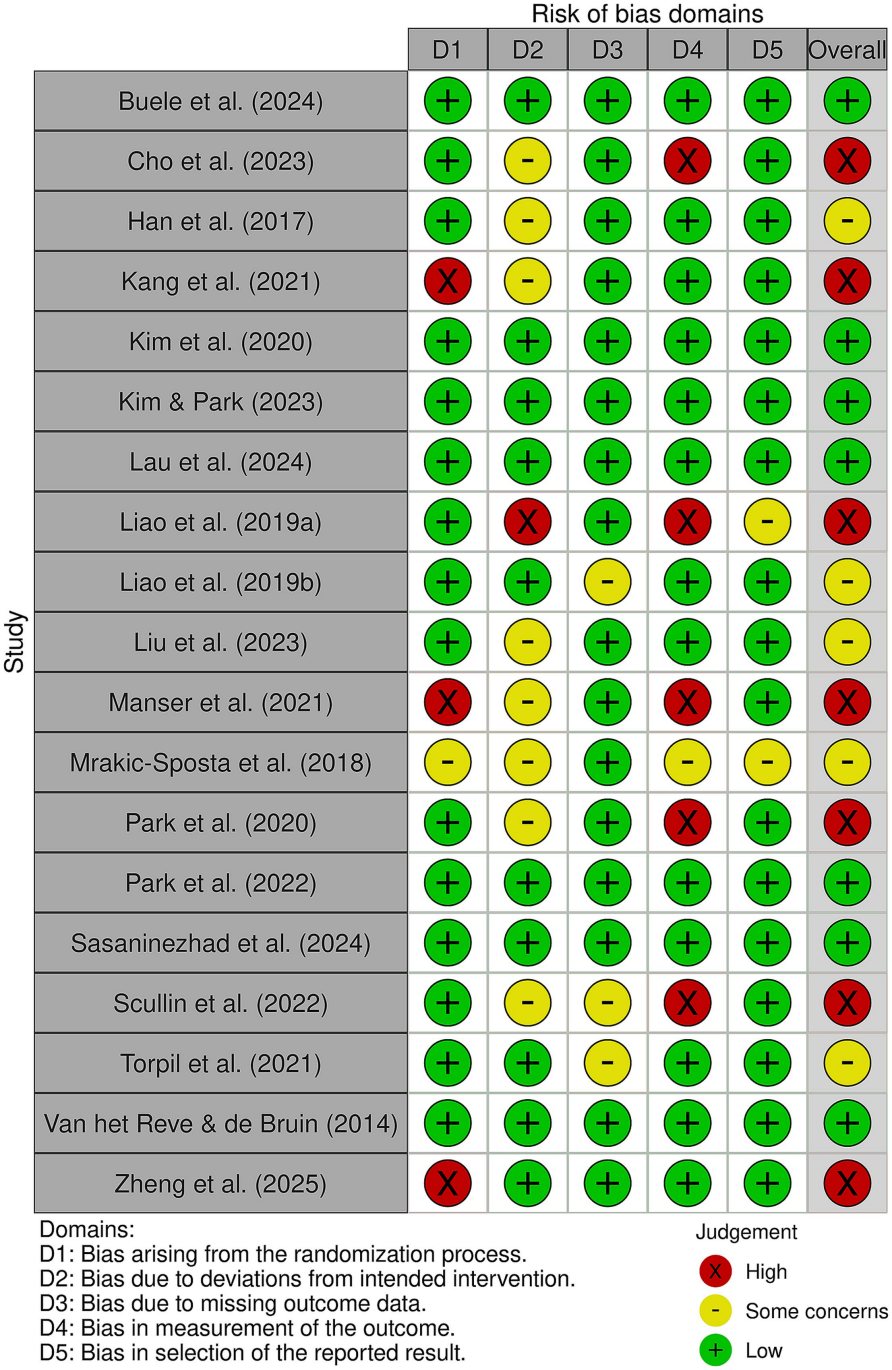
Risk of bias: traffic light graph.

Seven studies were rated as having a low risk of bias (38, 42–44, 51, 52, 55), five presented some concerns (40, 46, 47, 49, 54), and seven were classified as having a high risk of bias (39, 41, 45, 48, 50, 53, 56). This indicates an overall risk of bias across the included studies, as most presented some methodological concerns, while three were classified as having a high risk of bias. Figures 2, 3 summarize the risk of bias.

### 3.4 Characteristics of the studies

Among the 19 reviewed studies, interventions included cognitive rehabilitation combined with physical-cognitive training using VR, cognitive training with applications, and dual physical-cognitive training. All included studies used VR interventions combined with cognitive training, but differed in immersion level, dose, and specific components. Immersive VR studies using head-mounted displays and fully 3D environments typically implemented integrated cognitive training over 6–12 weeks (3 sessions/week; 30–45 min/session) and reported improvements in working memory, executive function, and IADL (51, 52, 54). Non-immersive VR trials using screen-based platforms and exergaming for physical-cognitive training were generally delivered 2–4 times/week for 6–12 weeks (40–60 min/session) and showed gains in cognitive flexibility, memory, attention, and balance (46, 47, 55). Overall program lengths ranged from 4 to 16 weeks with session frequencies of 2–5 per week and durations of 20–120 min; despite heterogeneity in modality and dosing, most VR protocols yielded cognitive and functional benefits, although some specific VR cognitive-motor programs.

### 3.5 Sample characteristics

This systematic review with meta-analysis encompassed a total population of 722 older people (63.5% female; mean age of 74.2 years), all of whom had a medical diagnosis of MCI. Sample sizes varied from 21 participants (44) to 156 participants (55), illustrating the variability of studies (Table 2).

**Table 2:** Selected studies on virtual reality-based cognitive training on cognitive function, instrumental activities of daily living, and depressive symptoms in older people with mild cognitive impairment.

Study	Country	Cluster (n)	Average age (years)	Types of intervention and control group	Volume training			Training intensity assessment	Cognitive function	Instrumental activities daily living	Depressive symptoms	Main results
					Weeks	Frequency (sessions/week)	Session duration (min)					
Buele et al. (38)	EC	EG: 17 CG: 17	77.35 years old; 58.8% females	EG: Cognitive training based on VR CG: Traditional cognitive training	6	2	40	Low	MoCA	IADL	GDS	MoCA: Control Arm (*p* < 0.001) Experimental Arm (*p* < 0.001) GDS: Control Arm (*p* < 0.001) Experimental Arm (*p* = 0.005) IADL Performance: Control Arm (*p* = 0.317) Experimental Arm (*p* = 0.102)
Cho et al. (39)	SK	EG: 50 CG: 50	71.24 years old; 68.3% females	EG: App BeauBrain Trainer CG: Usual ADL	16	5	90	Low	BeauBrain CST, K-ECog, SMCQ and AQS	B-ADL	GDS	BeauBrain CST: Cognitive Performance: Significant improvement in language domain scores (*p* = 0.019) Total Score: No significant difference (*p* = 0.297) Attention Domain: No significant difference (*p* = 0.694) Visuospatial Domain: No significant difference (*p* = 0.081) Memory Domain: No significant difference (*p* = 0.331) Executive Function Domain: No significant difference (*p* = 0.906) K-ECog: No significant difference (*p* = 0.797) B-ADL: No significant difference (*p* = 0.752) GDS: No significant difference (*p* = 0.957)
Han et al. (40)	SK	EG: 20 CG: 23	73.74 years old; 43.5% females	EG: USMART CG: Usual ADL	8	2	30	Low	WLMT, WLRT, WLRcT, SMCQ and MMSE	NR	GDS	WLRT: Experimental Arm: ↑ Improvement (*p* = 0.031) WLRcT: No significant difference (*p* = 0.229) SMCQ: No significant difference (*p* = 0.705) GDS: No significant difference (*p* = 0.799) MMSE: No significant difference (*p* = 0.118)
Kang et al. (41)	SK	EG: 23 CG: 18	74.51 years old; 73.9% females	EG: Usual therapy + cognitive training in a VR environment CG: Usual ADL	4	2	20–30	Low	MMSE and RCFT	NR	GDS	MMSE: No significant difference (*p* = 0.93) IADL: No significant difference (*p* = 0.27)
Kim et al. (42)	SK	EG: 16 CG: 16	69.94 years old; 87.5% females	EG: computer-based cognitive rehabilitation in addition to RehaCom cognitive rehabilitation CG: RehaCom cognitive rehabilitation	8	3	30	Low	ADAS-K-cog and MoCA-K	ADL and IADL	NR	ADAS-K-cog: EG: Z = −3.42, *p* = 0.001 (↑ Improvement) Both groups (EQ-5D-5L): No significant change MoCA-K: EG: Z = −3.53, *p* < 0.001 (↑ Improvement) Both groups (EQ-5D-5L): No significant change ADL: Both groups (EQ-5D-5L): No significant change IADL: Both groups (EQ-5D-5L): No significant change
Kim & Park (43)	SK	EG: 21 CG: 21	74.33 years old; 57.1% females	EG: Cognitive–physical dual-task training CG: Computerized cognitive training focusing on executive function	8	2	45	Low	EFPT-K and FAB	IADL	NR	EFPT-K EG vs. CG: EG ↑ Improvement in executive function Both groups ↔ Pre-intervention: *p* < 0.001 Post-intervention: *p* < 0.001 FAB: EG vs. CG: EG ↑ Improvement in frontal lobe function Both groups ↔ Pre-intervention: *p* < 0.001 Post-intervention: *p* < 0.001 K-IADL: EG vs. CG: EG ↑ Improvement in instrumental activities Both groups ↔ Pre-intervention: *p* < 0.001 Post-intervention: *p* < 0.001
Lau et al. (44)	TW	EG: 11 CG: 10	72 years old; 63.6% females	EG: tDCS + ICCT CG: Sham + ICCT	5	3	40	Low	MoCA, N-Back Task, CVVLT and TMT	NR	NR	MoCA: Experimental Arm ↑ (*p* = 0.002) Both groups (EQ-5D-5L): Control Arm ↑ (*p* = 0.014) TMT-A: Experimental Arm ↑ (*p* < 0.001) TMT-B: Experimental Arm ↑ (*p* = 0.002) Both groups (EQ-5D-5L): Control Arm ↑ (*p* = 0.012) CVVLT: Experimental Arm ↑ (*p* < 0.001) Both groups (EQ-5D-5L): Control Arm ↑ (*p* = 0.008) CVVLT: Experimental Arm ↑ (*p* < 0.001) Both groups (EQ-5D-5L): Control Arm ↑ (*p* = 0.003) N-back Task 1-back: Experimental Arm ↑ (*p* < 0.001) Both groups (EQ-5D-5L): Control Arm ↑ (*p* = 0.002) N-back Task 2-back: Experimental Arm ↑ (*p* < 0.001) Both groups ↔ QoL
Liao et al. (45)	TW	EG: 18 CG: 16	75.5 years old; 75% females	EG: VR-based physical and cognitive training CG: Combined physical and cognitive training	12	3	60	Low	TMT and SCWT			Executive Function: VR: ↑ in TMT-B (*p* = 0.032) VR: ↑ in Delta TMT (*p* < 0.001) Both groups ↔ Outcome Measure: Both groups ↔ SCWT-numbers (VR: *p* < 0.001, CPC: *p* = 0.002) Cognitive dual-task gait performance: VR: ↑ in cognitive DTCs of cadence (*p* = 0.018)
Liao et al. (46)	TW	EG: 18 CG: 16	75.5 years old; 61% females	EG: VR-based physical and cognitive training CG: Combined physical and cognitive training	12	3	60	Moderate	MoCA, CVVLT—Immediate Recall, CVVLT—Delayed Recall and Brain Activation	IADL	NR	MoCA: VR Arm ↑ (*p* < 0.001) CVVLT—Immediate Recall VR Arm ↑ (*p* < 0.001) CVVLT—Delayed Recall VR Arm ↑ (*p* = 0.002) IADL: VR Arm ↑ (*p* < 0.001) Group × Time Interaction (*p* = 0.006) Brain Activation (Prefrontal Cortex): VR Arm ↑ (*p* = 0.0015)
Liu et al. (47)	CH	TC: 17 EXER-TC: 16 CG: 17	74,6 75% female	EG: Tai Chi EXER-TC: Tai Chi exergaming CG: Usual ADL	12	3	50	Moderate	MoCA, TMT-A, TMT-B and SCWT	NR	NR	MoCA: EXER-TC vs. CG: *p* = 0.008 Experimental Arm: Improvement in cognitive function TMT-A: EXER-TC vs. CG: *p* < 0.001 Experimental Arm: Faster processing speed TC vs. CG: *p* = 0.006 TC: ↑ Faster processing speed TMT-B: EXER-TC vs. CG: *p* < 0.001 Experimental Arm: Improvement in cognitive flexibility TC vs. CG: *p* = 0.004 TC: ↑ Improvement in cognitive flexibility Δ TMT: EXER-TC vs. CG: *p* = 0.001 Experimental Arm: Decrease in cognitive load TC vs. CG: *p* = 0.007 TC: Decrease in cognitive load One-back test: EXER-TC vs. CG: *p* = 0.001 Experimental Arm: Improvement in working memory TC vs. CG: *p* = 0.005 TC: Improvement in working memory
Manser et al. (48)	CH	EG: 12 CG: 6	73.3 years old; 66.7% females	EG: Cognitive motor training based on exergames CG: Usual ADL	12	4	20	Low	NR	NR	DASS-21 -Stress	DASS-21 – Stress: No significant difference (*p* = 0.335)
Mrakic-Sposta et al. (49)	USA	EG: 5 CG: 5	72 years old; 60% females	EG: VR-based physical and cognitive training CG: Usual ADL	6	3	40–45	Low	MMSE, ROCFT, FAB, RAVLT_I, RAVLT_D, AM, TMT-A and VF	NR	NR	MMSE: *p* = 0.690 ROCFT: *p* = 0.690 FAB: *p* = 1.000 RAVLT_I: *p* = 0.421 RAVLT_D: *p* = 1.000 AM: *p* = 0.421 TMT-A: *p* = 0.310 VF: *p* = 0.095
Park et al. (50)	SK	EG: 18 CG: 17	75.8 years old; 44.4% females	EG: Cognitive rehabilitation based on MOTOCOG® CG: Traditional cognitive rehabilitation	6	5	30	Low	MoCA, TMT-A, TMT-B and Digit Span Test	NR	NR	MoCA: Experimental Arm: Significant improvement (*p* < 0.001) TMT-A: Experimental Arm: Significant improvement (*p* < 0.001) TMT-B: Experimental Arm: Significant improvement (*p* < 0.001) Digit Span Test – Forward: Experimental Arm: Significant improvement (*p* < 0.001) Digit Span Test – Backward: Experimental Arm: Significant improvement (*p* < 0.001)
Park (51)	SK	EG: 16 CG: 16	72.25 years old; 43.8% females	EG: Virtual shopping training CG: Waitlist training	8	2	30	Low	EFPT-K	K-IADL	NR	EFPT-K: ↑ Improvement in executive function (*p* < 0.001) K-IADL: ↑ Improvement in (*p* < 0.001)
Sasaninezhad et al. (52)	IR	EG: 20 CG: 20	70.3 years old; 62% females	EG: VR cognitive rehabilitation CG: Cognitive rehabilitation does not receive the VR	10	3	30	Low	WCST, Symbol Span Subtest and Digit Span Subtest	IADL	GDS	Depression Symptoms: Time main effect F(1.59, 60.26) = 18.68, *p* < 0.001 Interaction *F*(1.59, 60.26) = 26.33, *p* < 0.001 Group main effect *F*(1, 38) = 0.74, *p* = 0.396 Post-test (Intervention group) t(38) = −3.15, *p* = 0.003 Working Memory: Backward Digit Span (Post-test) VR Arm ↑ t(38) = 3.67, *p* < 0.001 Backward Digit Span (Follow-up) VR Arm ↑ t(38) = 5.78, *p* < 0.001 Forward Digit Span (Baseline to Post-test) VR Arm ↑ t(19) = −6.75, *p* < 0.001 Forward Digit Span (Baseline to Follow-up) VR Arm ↑ t(19) = −7.03, *p* < 0.001 Visual Working Memory: Symbol Span (Time main effect) *F*(2, 76) = 8.25, *p* = 0.001 Symbol Span (Interaction) F(2, 76) = 35.28, *p* < 0.001 Symbol Span (Follow-up) VR Arm ↑ t(38) = −0.62, *p* = 0.006 Control Group—Forward Digit Span: Baseline to Follow-up t(19) = 2.60, *p* = 0.017 Control Group—Backward Digit Span Baseline to Post-test t(19) = −3.90, *p* < 0.001 Baseline to Follow-up t(19) = −4.07, *p* < 0.001 IADL = ↑ Intervention group showed significantly higher levels of IADL scores at post-test (*p* < 0.001) and follow-up (*p* < 0.001) compared to control group Both groups = ↔ No significant difference at baseline (*p* = 0.396)
Scullin et al. (53)	USA	EG: 26 CG: 26	73.17 years old; 52% females	EG: Reminder App CG: Digital Recorder App	4	2	120–240	Low	PRMQ and Neuro-QoL	IADL	NR	IADL (Care Partner Report): Experimental Arm ↑ Improvement (*p* = 0.049) Overall improvement in prospective memory tasks: Experimental Arm ↑ Improvement (*p* < 0.001)
Torpil et al. (54)	TR	EG: 30 CG: 31	70.12 years old; 63.3% females	EG: Therapy CR + VR CG: Therapy CR	12	3	45	Low	LOTCA-G	NR	NR	CR Arm—CR+VR Arm (CR Arm ↑): Orientation (*p* < 0.05) Visual Perception (*p* < 0.05) Spatial Perception (*p* < 0.05) Motor Praxis (*p* < 0.001) Visuomotor (*p* < 0.05) Thinking Operation (*p* < 0.05) Memory (*p* < 0.001) Attention/Concentration (*p* < 0.05) Total Test Score (p < 0.05)
van het Reve & de Bruin (55)	CH and DE	EG: 74 CG: 82	81.1 years old; 66.2% females	EG: SBC-Strength Balance Cognitive CG: SB- Strength Balance	12	2	60	Low	TMT-A and TMT-B	NR	NR	TMT-A and TMT-B: Experimental Arm ↑: Significant improvement over time (*p* < 0.001)
Zheng et al. (56)	CN	EG: 33 CG: 33	80.88 years old; 64% females	EG: Virtual reality-based ADL training CG: ADL.	12	2	45	Low	MMSE and GDS-15	FIM, IADL and BI	GDS	FIM: Experimental Arm ↑ Both groups (EQ-5D-5L): *p* < 0.001 BI: Experimental Arm ↑ Both groups (EQ-5D-5L): *p* < 0.001 IADL: Experimental Arm ↑ Both groups (EQ-5D-5L): *p* = 0.001 MMSE: Experimental Arm ↑ Both groups (EQ-5D-5L): *p* < 0.001 GDS-15: Experimental Arm ↓ Both groups (EQ-5D-5L): *p* = 0.017

ADAS-K-cog: Korean version of the Alzheimer’s Disease Assessment Scale-Cognitive Subscale; ADL: Activities of Daily Living; AM: Autobiographical Memory; AQS: Attention Questionnaire Scale; B-ADL: Bayer Activities of Daily Living; BeauBrain CST: BeauBrain Cognitive Training Software Test; BI: Barthel Index; CG: Control Group; CH: Switzerland; CN: China; CR: Cognitive Rehabilitation; CVVLT: Chinese Version of the Verbal Learning Test; DASS-21: Depression Anxiety Stress Scales-21 Items; DE: Germany; DTC: Dual Task Costs; EC: Ecuador; EFPT-K: Executive Function Performance Test-Korean version; EG: Experimental Group; EQ-5D-5L: EuroQol 5 Dimensions 5 Levels; EXER-TC: Tai Chi Exergaming; FAB: Frontal Assessment Battery; FIM: Functional Independence Measure; GDS: Geriatric Depression Scale; GDS-15: Geriatric Depression Scale-15 items; IADL Lawton y Brody: Instrumental Activities of Daily Living; ICCT: Individualized Computerized Cognitive Training; IR: Iran; K-IADL: Korean Instrumental Activities of Daily Living; K-ECog: Korean Everyday Cognition; LOTCA-G: Loewenstein Occupational Therapy Cognitive Assessment-Geriatric version; MCI: Mild Cognitive Impairment; MMSE: Mini-Mental State Examination; MoCA: Montreal Cognitive Assessment; MoCA-K: Korean version of the Montreal Cognitive Assessment; MOTOCOG®: A computerized cognitive rehabilitation program; N-back Task: 1 or 2 steps back working memory tasks; Neuro-QoL: Quality of Life in Neurological Disorders; NR: Not Reported; PRMQ: Prospective and Retrospective Memory Questionnaire; RCT: Randomized Controlled Trial; RCFT: Rey-Osterrieth Complex Figure Test; RAVLT_I: Rey Auditory Verbal Learning Test-Immediate Recall; RAVLT_D: Rey Auditory Verbal Learning Test-Delayed Recall; RehaCom: A computerized cognitive rehabilitation software; ROCFT: Rey-Osterrieth Complex Figure Test; SCWT: Stroop Color and Word Test; SK: South Korea; SMCQ: Subjective Memory Complaints Questionnaire; SPPB: Short Physical Performance Battery; SBC: Strength Balance Cognitive; SB: Strength Balance; SAGE: Self-Administered Gerocognitive Examination; tDCS: Transcranial Direct Current Stimulation; TC: Tai Chi; TMT: Trail Making Test; TMT-A: Trail Making Test Part A; TMT-B: Trail Making Test Part B; TR: Turkey; TW: Taiwan; USA: United States of America; USMART: Ubiquitous spaced retrieval-based memory Advancement and Rehabilitation Training; VF: Verbal Fluency; VR: Virtual Reality; WCST: Wisconsin Card Sorting Test; WLMT: Word List Memory Test; WLRT: Word List Recognition Test; WLRcT: Word List Recall Test.

### 3.6 Cognitive function

The results of the analysis showed the following for the MoCA, the random model indicated an effect size of 0.87 (95% CI: 0.29 to 1.45), with a *p*-value of 0.003, which was statistically significant. The I^2^ value was 69.1%, suggesting high heterogeneity among the studies. Additionally, Egger’s test yielded a p-value of 0.01 for MoCA, which may suggest small-study effects; however, this finding should be interpreted cautiously given the limited number of studies included. The weighted range (WR) was 31.3–36.8%. For TMT-A, the random model showed an effect size of 1.10 (95% CI: −0.46 to 2.67), with a *p*-value of 0.16, meaning the result was not statistically significant. The I^2^ value was 92.1%, indicating very high heterogeneity. Egger’s test yielded a p-value of 0.00, which may be compatible with small-study effects; however, this result should be interpreted cautiously because the number of studies was small and between-study heterogeneity was very high. The weighted range was 16.3–21.6%. Finally, for TMT-B, the fixed model showed an effect size of 1.12 (95% CI: 0.71 to 1.53), with a highly significant p-value of 0.000. The I^2^ value was 0.00%, indicating low heterogeneity among the studies. Egger’s test yielded a p-value of 0.74. Although this does not suggest clear small-study effects, the result should still be interpreted cautiously given the limited number of studies available. The weighted range was 22.8 to 25.7%. The number of fail-safe for Hedges’s studies observed in the cognitive function for MOCA was 2, TMT-A 1 and TMT-B 0. These results are presented in a funnel plot following the forest plots of the tests. The individual studies reported improvements in specific cognitive domains following virtual reality, exergaming, and cognitive training interventions. Han et al. (40) found significant improvement in word list recall (WLRT) in the experimental group (*p* = 0.031), although other memory measures showed no significant changes. Kim et al. (42) observed significant enhancement in ADAS-K-cog scores after computer-based cognitive rehabilitation (*p* = 0.001). Kim & Park (43) reported improvements in executive function and frontal lobe function measured by EFPT-K and FAB (*p* < 0.001) following cognitive–physical dual-task training. Sasaninezhad et al. (52) found significant gains in working memory and visual working memory with VR cognitive rehabilitation, including backward and forward digit span and Symbol Span (*p* < 0.001). Scullin et al. (53) demonstrated that a reminder app led to better prospective memory performance compared to a digital recorder app (*p* < 0.001; Supplementary Figures 1–3, 6–8; Supplementary Table 1).

### 3.7 Instrumental activities of daily living

The result for IADL showed a random model with an effect size of 1.16 (95% CI: 0.67 to 1.64), with a highly significant *p*-value of 0.000. The I^2^ value was 70.8%, indicating high heterogeneity among the studies. Additionally, Egger’s test yielded a *p*-value of 0.00 for IADL, which may suggest small-study effects; however, this finding should be interpreted cautiously given the limited number of studies and the substantial heterogeneity observed. The weighted range was 57.9 to 65.7%. The number of fail-safe for Hedges’s studies observed in the IADL test was 2. These results are presented in a funnel plot following the forest diagrams of the tests mentioned (Supplementary Figures 4, 9; Supplementary Table 1).

### 3.8 Depressive symptoms

The result for Geriatric Depression Scale (GDS) Showed a random model with an effect size of 1.05 (95% CI: 0.39 to 1.71), with a statistically significant *p*-value of 0.002. The I^2^ value was 75.5%, indicating high heterogeneity among the studies. Additionally, Egger’s test yielded a p-value of 0.00 for GDS, which may suggest small-study effects; however, this result should be interpreted cautiously given the limited number of studies included and the high heterogeneity across trials. The weighted range was 37.8 to 38.2%. The individual studies reported effects on depressive symptoms using instruments other than the GDS. Manser et al. (48) found no significant difference in stress levels measured by the DASS-21 between the cognitive motor training group and control (*p* = 0.335). Zheng et al. (56) observed a significant reduction in depressive symptoms in the experimental group after virtual reality-based ADL training, as measured by the GDS-15 (*p* = 0.017), indicating improved mood and emotional well-being. The number of fail-safe for Hedges’s studies observed in depressive symptoms in the GDS test was 1. These results are presented in a funnel plot following the forest plots of the tests mentioned (Supplementary Figures 5, 10; Supplementary Table 1).

### 3.9 Certainty of evidence

The certainty of evidence for VR cognitive training was moderate for cognitive function, IADL, and depressive symptoms. Although the included studies were RCTs, concerns related to risk of bias reduced the certainty of the evidence. Overall, the findings suggest potential benefits in older people with MCI; however, their interpretation should remain cautious given the methodological limitations and heterogeneity across studies (Table 3).

**Table 3:** Assessment of methodological quality using the GRADEpro tool.

Certainty of evidence							N° of patients		Effect		Certainty	Importance
N° of studies	Study design	Risk of bias	Inconsistency	Indirect evidence	Vagueness	Other considerations	NIBS and cognitive training	Active control group	Relative (95% CI)	Absolute (95% CI)		
Cognitive Function												
5	RCT	Serious	It’s not serious	It’s not serious	It’s not serious	None	102/185 (55.1%)	83/185 (44.9%)	Not estimable		+++ Moderate	Important
Instrumental activities of daily living												
7	RCT	Serious	It’s not serious	It’s not serious	It’s not serious	None	139/280 (49.6%)	141/280 (50.4%)	Not estimable		+++ Moderate	Important
Depressive symptoms												
4	RCT	Serious	It’s not serious	It’s not serious	It’s not serious	None	93/181 (51.4%)	88/181 (48.6%)	Not estimable		+++ Moderate	Important

NIBS, noninvasive brain stimulation; RCT, Randomized clinical trial.

### 3.10 Adverse effects and adherence

The studies in this systematic review with meta-analysis reported an 84.2% adherence rate and no adverse effects, suggesting that the interventions were well tolerated and feasible for older people with MCI. These findings support their potential for broader implementation in similar populations.

## 4 Discussion

### 4.1 Cognitive functions

The present meta-analysis suggests that VR-based cognitive training may improve cognitive performance across some domains in older people with MCI. Statistically significant improvements were observed in MoCA scores and TMT-B performance, indicating potential benefits in global cognition and executive functioning. In contrast, changes in TMT-A scores were not significant, suggesting that the effects may not extend consistently across all cognitive domains. Consistent with these findings, Ghous et al. (58) reported comparable improvements in global cognition among older people following both task-oriented circuit training and VR-based interventions, as reflected by significant MoCA score increases (*p* < 0.05). Similarly, Yang et al. (59), in a meta-analysis of 18 studies, found that VR interventions yielded significant benefits in memory, attention, processing speed, and executive function among older peoples with MCI. Complementary evidence from Vásquez-Carrasco et al. (24) revealed that VR-based interventions also enhanced cognitive performance and quality of life in individuals with Alzheimer’s disease.

Further support is provided by Rogers et al. (60), who examined integrated motor and cognitive VR training in adults undergoing subacute stroke rehabilitation. Following a four-week program, the intervention group exhibited significant cognitive improvements (*p* < 0.001), which persisted during follow-up assessments. Likewise, Cao et al. (61) demonstrated that artificial intelligence–assisted and VR-based training protocols produced greater improvements in cognitive functioning relative to control groups, with significant gains in attention (*p* = 0.006), orientation (*p* = 0.01), and memory (*p* = 0.02). Collectively, these findings suggest that VR-based cognitive interventions may offer potential cognitive benefits across different populations, although the magnitude and consistency of these effects appear to vary according to the clinical context, intervention characteristics, and study quality. The immersive nature of VR engages multiple sensory modalities, promoting participation in complex, ecologically valid tasks, while non-immersive VR formats encourage cognitive engagement through interactive feedback. Both modalities have been associated with improvements in key cognitive domains, including working memory, attention, executive function, and decision-making (11, 26).

### 4.2 Instrumental activities of daily living

The meta-analysis reported that IADL had significant improvements in favor of VR-based cognitive training (*p* = 0.001). Similar results were reported by a RCT that implemented VR training in post-stroke individuals, where IADL increased significantly (*p* < 0.0001) (62). Different results were reported by a RCT where the experimental group used a portable VR training device with activity-based training, the IADL score did not show significant results (*p* = 0.979) (63). Similar results were reported by an RCT that implemented VR training to stimulate cognition in people with Alzheimer’s disease, showing no significant effects on IADL (*p* = 0.905) (64). A systematic review reported evaluating 26 articles, they reported that some technology-based cognitive interventions showed improvements in IADL, highlighting their functional potential (65). Another RCT found that intervening in IADLs with rehabilitation and health education did not result in significant improvements (66). These results suggest that although VR may have a positive impact in some contexts, its effectiveness in improving IADL varies depending on specific treatment conditions and approaches. VR-based cognitive training, both immersive and non-immersive, enhances IADL by providing motivating environments that facilitate repetitive practice of functional tasks. While immersive VR offers fully engaging 3D experiences, non-immersive VR supports skill practice through screen-based or exergaming platforms, promoting neuroplasticity and transfer of skills to real-life situations (66–68).

### 4.3 Symptoms of depression

The meta-analysis revealed that VR-based cognitive training was associated with a significant reduction in depressive symptoms, as measured by GDS (*p* = 0.002). These findings align with results from RCTs. For instance, in a study involving post-stroke patients, the group undergoing VR intervention showed a significant decrease in depressive symptoms (*p* < 0.01), particularly when the training was integrated with neurological rehabilitation, contributing to mood improvement and lower depression scores (62). A systematic review reported evaluating 9 articles, they reported that VR interventions showed positive effects on depressive symptoms and general well-being in older people, especially those with MCI (15). However, other RCTs report mixed outcomes. In a trial evaluating VR as a treatment for adults with obsessive-compulsive disorder, reductions in Beck Depression Inventory-II scores were observed in both the VR and control groups, with no statistically significant differences between them (*p* = 0.47) (69). Similarly, a study involving older people experiencing dizziness found no significant intergroup differences in GDS scores following VR training (*p* = 0.302) (70).

These heterogeneous results suggest that the impact of VR-based cognitive training on depressive symptoms may depend on several factors, including the clinical population, intervention design, and contextual variables. Despite this variability, both immersive and non-immersive VR offer potential cognitive benefits. Immersive VR creates fully engaging environments (73), while non-immersive VR promotes interaction through screen-based or exergaming platforms (74). Together, these approaches foster engagement, physical activity, social interaction, and cognitive stimulation factors known to support emotional regulation and enhance well-being (18, 71, 72).

### 4.4 Limitations and strengths

This systematic review and meta-analysis present several limitations. First, the included studies showed a mixed risk-of-bias profile, which may affect the internal validity of the pooled findings. Second, substantial heterogeneity was observed across several outcomes, particularly cognitive function, IADL, and depressive symptoms, limiting the consistency of the combined estimates. Third, the included interventions varied considerably in immersion level, technological format, treatment dose, and comparator conditions, which may have contributed to between-study variability. In addition, quantitative synthesis was restricted to the most consistently reported outcomes, while other relevant measures were summarized narratively because of limited comparability. Several pooled analyses also included a relatively small number of studies, reducing the stability of publication-bias assessments and robustness estimates. Finally, all studies assessed IADL using self-report or informant-report questionnaires, which may not fully capture functional performance.

Despite these limitations, this study also has notable strengths. It included randomized controlled trials and assessed a broad spectrum of VR-based cognitive interventions, offering an integrated perspective on their potential relevance for older people with MCI. In addition, the review examined clinically meaningful domains, including cognitive function, depressive symptoms, and IADL performance. Although the pooled analyses suggested beneficial effects in these outcomes, such findings should be interpreted with caution given the risk of bias, heterogeneity, and variability in intervention protocols across studies.

### 4.5 Practical applications

This systematic review with meta-analysis suggests that VR-based cognitive training may represent a feasible and potentially useful intervention for older people with mild cognitive impairment. VR provides immersive and task-oriented environments that may support global cognition, executive function, instrumental activities of daily living, and depressive symptoms. Its ecological characteristics may facilitate transfer to daily functioning, while the relatively high adherence rates and absence of reported adverse effects indicate that these interventions are generally well tolerated. Implementation in clinical and community-based settings should be considered cautiously until more methodologically robust evidence becomes available.

### 4.6 Clinical applications

The observed cognitive and functional findings suggest that VR-based cognitive training could be considered as a complementary component within multidisciplinary rehabilitation programs for older people with MCI. Interventions combining cognitive and physical components, such as VR-based physical-cognitive training and exergaming, may be particularly promising for improving executive function, cognitive flexibility, and functional independence. However, their clinical integration should remain guided by patient characteristics, intervention feasibility, and the still moderate certainty of the available evidence.

### 4.7 Epidemiological applications

From a public health perspective, VR-based cognitive training may have potential to contribute to delaying cognitive decline and reducing functional dependence in aging populations. Given the high prevalence of MCI and its role as a risk factor for dementia, these interventions could become relevant within preventive and rehabilitative strategies aimed at promoting healthy aging. However, conclusions regarding large-scale implementation remain premature, particularly in view of the limited evidence on long-term effectiveness, cost-effectiveness, and real-world applicability.

## 5 Conclusion

Interventions involving VR-based cognitive training were associated with improvements in cognitive function, instrumental activities of daily living, and depressive symptoms in older people with mild cognitive impairment. These findings suggest that VR may represent a promising tool for cognitive rehabilitation and functional support in this population. However, the results should be interpreted with caution due to the moderate certainty of the evidence, the heterogeneity across studies, and the methodological limitations identified in several trials. Further high-quality randomized controlled trials are needed to clarify the magnitude, consistency, and clinical applicability of these effects.

## Data Availability

The original contributions presented in the study are included in the article/Supplementary material, further inquiries can be directed to the corresponding authors.
