# Supplementary material for: Effects of virtual reality-based cognitive training on cognitive function, instrumental activities of daily living, and depressive symptoms in older people with mild cognitive impairment: a systematic review with meta-analysis of randomized controlled trials
Source: Front Neurol. 2026 Apr 13;17:1728495. doi: 10.3389/fneur.2026.1728495 (PMC13111015; doi:10.3389/fneur.2026.1728495)
Supplement: Supplementary file 1 [file Table_1.docx]

Supplementary Material

## Supplementary Figures


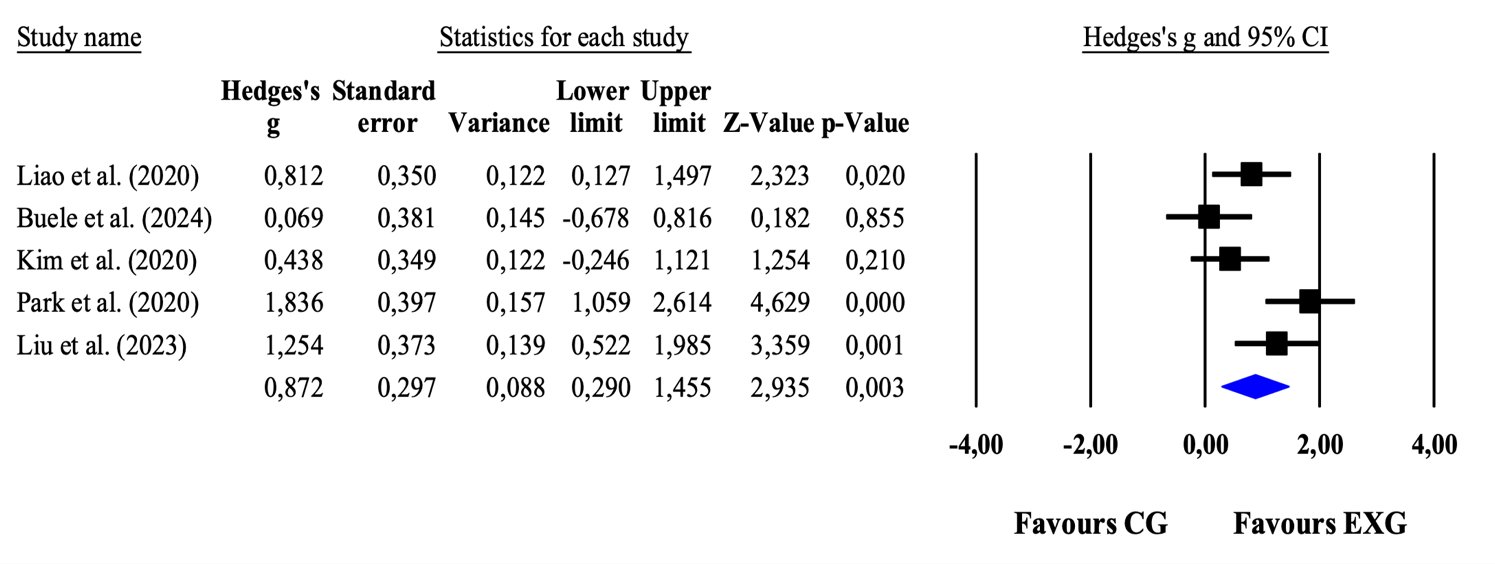
**Supplementary Figure 1.** Forest plot of changes in MoCA in 185 participating in 102 compared with 83 assigned as controls. Values shown are effect sizes (Hedges' g) with 95% confidence intervals (CI). The size of the squares plotted reflects the statistical weight of each study.

**
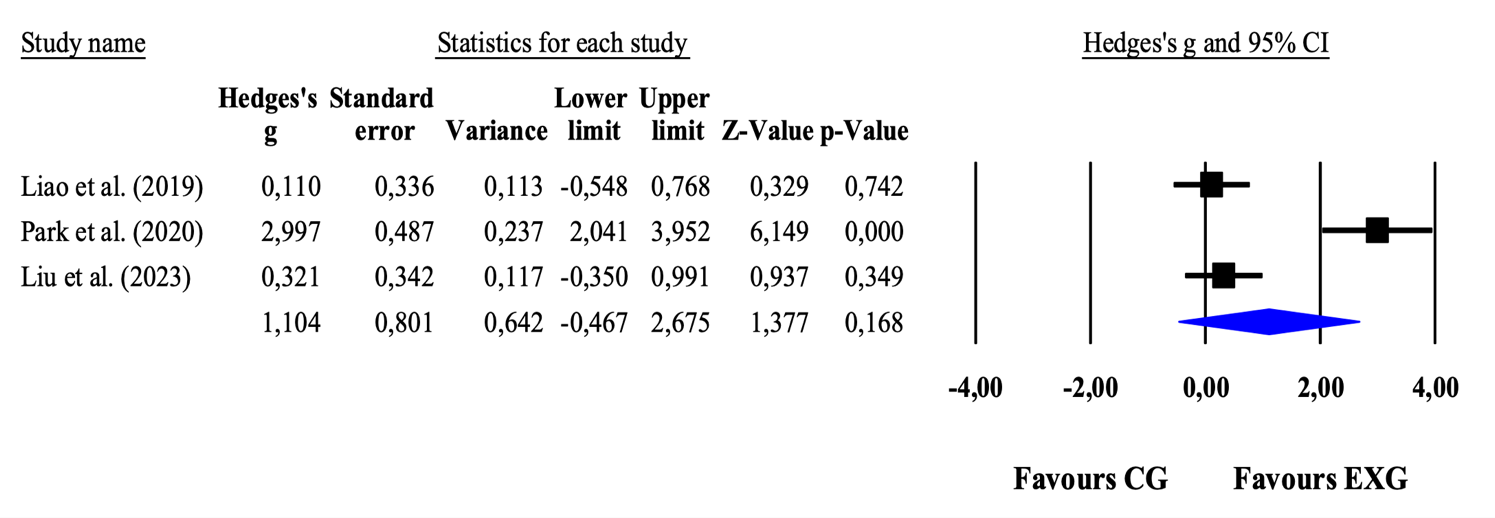
**

**Supplementary Figure 2.** Forest plot of changes in TMT-A in 119 participating in 69 compared with 50 assigned as controls. Values shown are effect sizes (Hedges' g) with 95% confidence intervals (CI). The size of the squares plotted reflects the statistical weight of each study.

**
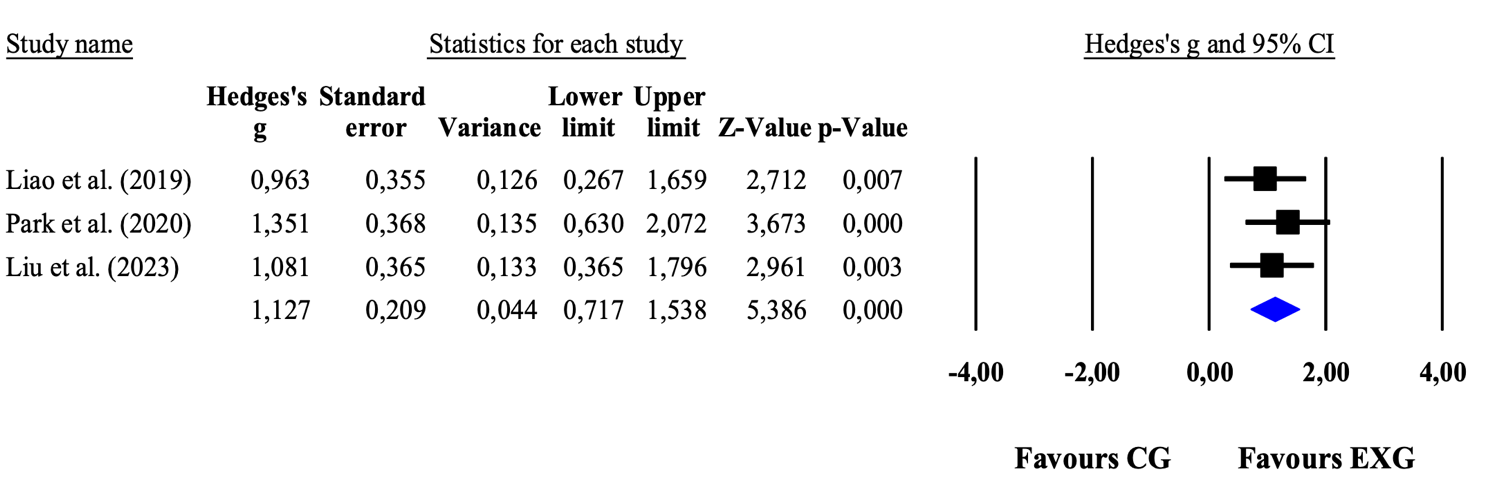
**

**Supplementary Figure 3.** Forest plot of changes in TMT-B in 119 participating in 69 compared with 50 assigned as controls. Values shown are effect sizes (Hedges' g) with 95% confidence intervals (CI). The size of the squares plotted reflects the statistical weight of each study.


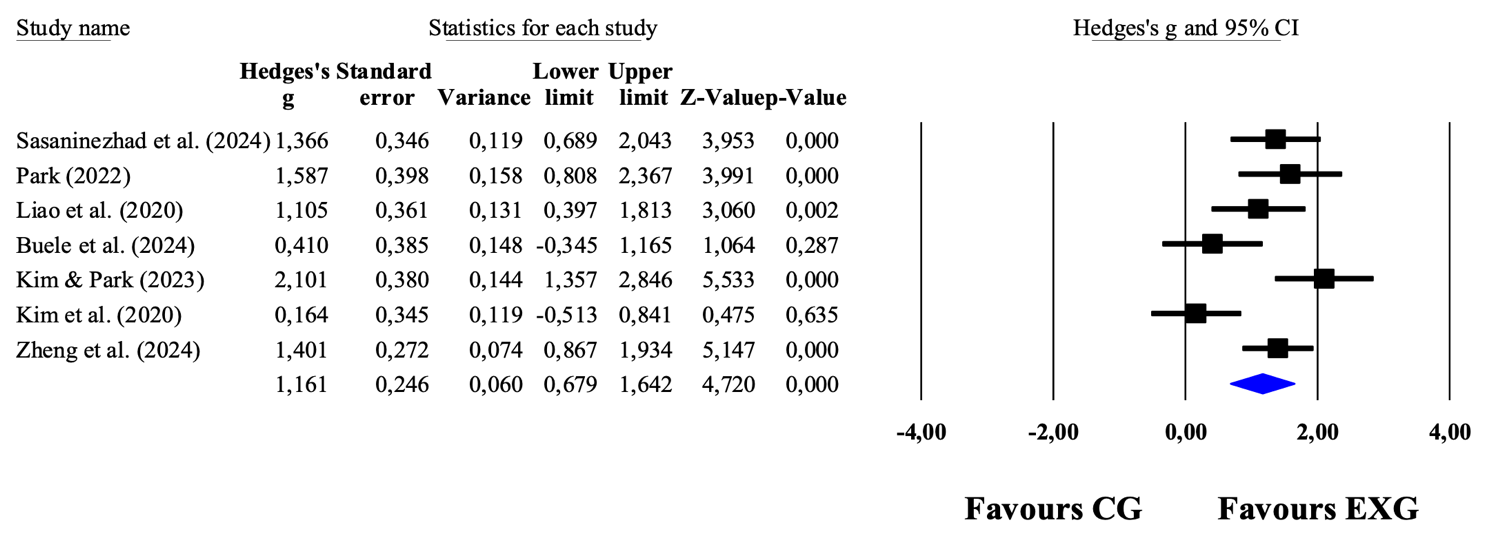


**Supplementary Figure 4.** Forest plot of changes in IADL in 280 participating in 139 compared with 141 assigned as controls. Values shown are effect sizes (Hedges' g) with 95% confidence intervals (CI). The size of the squares plotted reflects the statistical weight of each study.


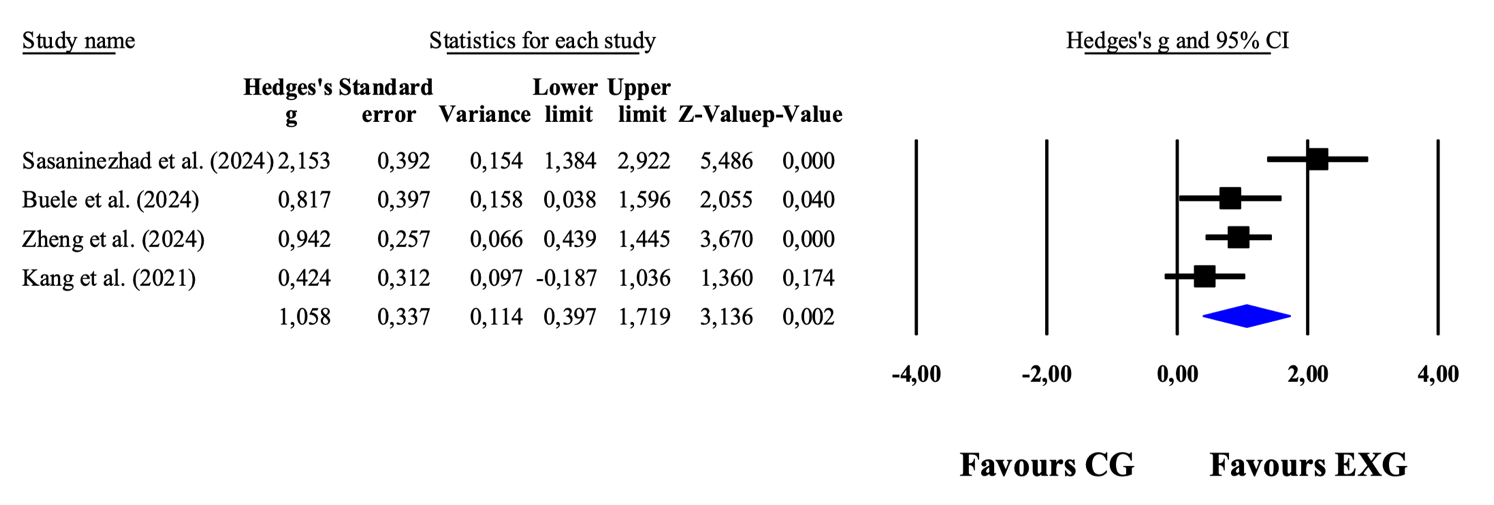


**Supplementary Figure 5.** Forest plot of changes in GDS in 181 participating in 93 compared with 88 assigned as controls. Values shown are effect sizes (Hedges' g) with 95% confidence intervals (CI). The size of the squares plotted reflects the statistical weight of each study.

**Supplementary Figure 6.** Funnel plot of studies reporting MoCA outcomes. The plot shows substantial heterogeneity among the included studies (I² = 68%; p = 0.01), indicating considerable variability in effect estimates beyond what would be expected by chance.

**Supplementary Figure 7.** Funnel plot for the TMT-A test. The wide dispersion of study estimates demonstrates very high heterogeneity across studies (I² = 92%; p = 0.000), suggesting marked between-study variability.

**Supplementary Figure 8.** Funnel plot of studies reporting TMT-B outcomes. The plot indicates very low heterogeneity among the included studies (I² = 0.0%; p = 0.74), suggesting minimal variability in effect estimates beyond chance.

**Supplementary Figure 9.** Funnel plot of studies reporting IADL outcomes. The plot indicates substantial heterogeneity among the included studies (I² = 70%; p = 0.02), suggesting considerable variability in effect estimates beyond chance.

**Supplementary Figure 10.** Funnel plot of studies reporting GDS outcomes. The plot indicates high heterogeneity among the included studies (I² = 75%; p = 0.02), suggesting substantial variability in effect estimates beyond chance.

## Supplementary Table

| **Outcome** | **Effect Size (SMD)** | **p-value** | **Heterogeneity (I²)** | **Publication Bias** | **Certainty (GRADE)** |
| --- | --- | --- | --- | --- | --- |
| Cognitive function | 0.87 (95% CI: 0.29–1.45) | p = 0.003 | 69.1% (High) | Egger’s test p = 0.01 | Moderate |
| Instrumental ADL | 1.16 (95% CI: 0.67–1.64) | p < 0.001 | 70.8% (Moderate) | Egger’s test p < 0.001 | Moderate |
| Depressive symptoms | 1.05 (95% CI: 0.39–1.71) | p = 0.002 | 75.5% (High) | Egger’s test p < 0.001 | Moderate |

ADL: Activities of Daily Living; SMD: Standardized Mean Difference.
